# Rare case of Alstrom syndrome with empty sella and interfamilial presence of Bardet-Biedl phenotype

**Published:** 2009

**Authors:** D Catrinoiu, C.M. Mihai, L Tuta, R Stoicescu, A Simpetru

**Affiliations:** * “Ovidius” University, Faculty of Medicine

**Keywords:** Alstrom syndrome, empty sella, Bardet-Biedl syndrome, ciliopathies

## Abstract

**Alstrom syndrome** is an extremely rare, autosomal recessive genetic disorder characterized by a group of signs and symptoms including infantile onset dilated cardiomyopathy, blindness, hearing impairment/loss, obesity, diabetes, hepatic and renal dysfunction. Since the first description of the syndrome in 1959, there have never been reported cases of Alstrom syndrome with the occurrence of the Bardet-Biedl syndrome in their relatives, this case suggesting a close genetic link between these two ciliopathies. The presence of empty sella seems to be a rare morphologic finding in Alstrom syndrome although it has been documented in few Bardet-Biedl cases.

**Case presentation**:

We report a case of a 20 -year-old caucasian male with hearing and visual loss, short stature, insulin resistant diabetes, dilated cardiomyopathy, hepatic and renal dysfunction, hypertension, and alopecia. By studying his family medical records we identified two relatives with suggestive clinical findings for Bardet –Biedl syndrome.

**Conclusion**:

Analyzing the clinical traits of these patients we found that retinopathy, nephropathy and central obesity were present in all patients, suggesting a main anomaly in ciliary function controlling photoreception, renal and metabolic processes. The occurrence of similar clinical cases within a family further demonstrates the existence of a common pathologic cilliary mechanism, a genetic basis of phenotypic variability in seemingly monogenic disease and a functional link between rare disorders and common traits with overlapping clinical manifestations. Genetic studies in such patients may provide new data regarding the consequences of defective cilia and a possible identification of new gene mutations.

## Introduction

Alstrom syndrome is caused by mutations in a gene of unknown function (*ALMS1*) and it is characterized by several reminiscent phenotypes of Biedl-Bardet syndrome, including retinal degeneration, obesity and diabetes. ALMS1 protein is located near the centrosomes and close to the base of the cilia. In fibroblasts with disrupted ALMS1, the primary cilia and the microtubule cytoskeleton appear to be normal, suggesting that the ALMS phenotype results from the impaired ciliary’s function rather than from the abnormal ciliary’s structure. (Hearn et al. 2005). Bardet–Biedl syndrome (BBS) is a rare human genetic disorder characterized by obesity, retinal dystrophy, renal anomalies, hypogenitalism, polydactyly, and cognitive deficits. BBS is a heterogeneous disorder that has been identified to have 12 causative genes (*BBS1–12*). Although the precise functions of the BBS proteins are still unresolved, numerous studies in different model systems have implicated the BBS proteins in cilia function. Seven of the most evolutionarily conserved BBS proteins (BBS1, 2, 4, 5, 7, 8, and 9) form a stable complex, called the BBSome, may mediate vesicular transport to the cilium [**1**]. The diagnosis of these syndromes is still mainly based on careful clinical examination. Studies on recessive diseases in Romania are constrained by low consanguinity rate and small number of children, which prevents statistical significance of the clinical and genetic results for each family.

## Case presentation

The patient with Alstrom syndrome is a 20-year-old son of non-consanguineous parents, and he has a healthy, unafected brother. The patient was born by vaginal delivery after a 40-week uncomplicated pregnancy; his birth weight was 2.9 kg. During his first year of life increased weight gain, progressive and worsening pathological ocular signs were noted: nystagmus, convergent strabismus, photophobia (at 12 months), reduced visual acuity. During evolution his diagnosis was „tapeto-retinal degeneration”. The first sign, nystagmus, was noted at the age of 4 months, then he developed progressive retinal distrophy and blindness at 10 years old. Infantile obesity (17 kgs at the age of 11 months) was also noted and progressed while aging. Sensorineural hearing impairment was noted at 15 years old and type 2 diabetes mellitus was diagnosed at 16 years old. From that point forward, he has been on insulin therapy, in different protocols, with 3 of 4 shots/day. In order to obtain a good glycemic control the needed dose of insulin was more than 200 IU/day. At this point, diabetes mellitus was considered the result of the resistance to the action of insulin. He never developed ketosis or diabetic keto-acidosis (DKA). While a child, he was evaluated with thyroid and metabolic dysfunctions and thyroid disorders so, lysosomal storage diseases and aminoacidopathies were excluded. While growing, several laboratory tests showed hyperglycemia, hypertriglyceridemia, hypercholesterolemia and increased liver transaminases. In the following years there was a progressive worsening of the metabolic functions. 

We investigated the systems and organs known to be affected in this disease. The ophthalmologic evaluation showed diffuse retinal dystrophy with dispersed pigment accumulations and bilateral pigmented macular scarring. The audiometric examination showed bilateral symmetric hypoacusia. Abdominal ultrasound showed hepatomegaly and steatosis. Systolic dysfunction of the left ventricle was noted on echocardiography (long parasternal axis showed diffuse hypokinesia with mitral valve failure). Using Tissue Doppler Application, longitudinal velocity shortening and pulmonary hypertension of 65-70 mmHg were also found. The patient had fasting hyperglycemia (16.5mmol/L), HbA1c (19.9%), basal insulin (51mU/L) and glycosuria (65.3mmol/L). The tests for anti-islet cell antibodies (ICA) and antibodies to glutamic acid decarboxilase (GAD-Abs) were negative.

Because Growth Hormone deficiency and Alstrom Syndrome share some clinical and metabolic features, we studied the GH-IGF1 (Insulin Growth Factor 1) axis, using MRI techniques and dynamic tests (insulin tolerance test). The MRI study of the diencephalic and pituitary region was suggestive for the diagnosis of empty sella. We found normal TSH (Thyroid Stimulating Hormone), fT3 (free triiodothyronine), fT4 (free thyroxine), and negative results for anti-thyroid auto-antibodies. Growth hormone stimulatory tests were taken and insulin provocative test revealed a severe GH deficiency in this patient, defined by a peak response to insulin-induced hypoglycemia less than 3ng/dL and IGF1 concentrations less than –2SDS.The values were: GH1 – 0.18ng/mL, GH2 – 0.49ng/mL (after the stimulation with insulin 0,4IU/kg/dose), IGF-1 – 49ng/mL (N :116-356ng/ml).

In conclusion, multiple pituitary hormone deficiency (MPHD) wasn’t found and we only noticed severe GH deficiency. The presence of empty sella seems to be a rare morphologic finding in Alstrom syndrome though it was described in patients with Bardet-Biedl syndrome [**2**]. Decreased serum levels of GH, which act on cardiac myocytes primarily through IGF-1, are associated with impaired myocardial growth and function, which can be improved with restoration of GH/IGF-1 homeostasis. This effect of GH on cardiac tissue may explain why the patient had multiple episodes of cardiac decompansation due to severe cardiomyopathy. In animal models and among human adults with heart failure attributed to dilated cardiomyopathy, treatment with GH results in acquisition of left ventricular (LV) mass and improved LV function, through a combination of mechanisms. Future studies are needed to assess if the substitution therapy with Recombinant Growth hormone is cost–effective and risk-free in patients with Alstrom Syndrome and severe insulin resistance. 

By studying his family’s medical records we identified two relatives with suggestive clinical findings for Bardet-Biedl syndrome. The two children (Alstrom patient’s relatives), brother (5 years old) and sister (15 years old) were clinically diagnosed with Bardet-Biedl syndrome based on the presence of rod-cone dystrophy, postaxial polydactyly (hexadactyly fifth finger in one hand and foot), central obesity, cognitive impairment, hypogonadism and hypogenitalism, mental retardation. Parents denied consanguinity in the family and were questioned about the onset of visual problems, renal disease, diabetes mellitus, and deafness. Nystagmus was present at the age of 3 and 4, respectively. We used direct ophthalmoscopy and electroretinograms for these two patients and ocular fundus has shown a waxy pale optic disc, atrophy of the choroids, narrow or obliterated vessels, and atypical pigmentation in the periphery suggesting the presence of retinitis pigmentosa. Electroretinogram recordings showed no rod responses to dim blue light. The general systemic examination comprised measurements of height and weight. The presence of ataxia and paraplegia was estimated by inspection of the gait and by finger to nose test. Both had normal perceptive hearing. Obesity, according to age and gender, defined by a body mass index (BMI) >28kg/m2, was found in the girl patient. She was also diagnosed with paresis of her lower extremities caused by spinal stenosis, while her affected brother had no history of paresis. She also presented a positive oral glucose tolerance test, but after a program of diet and exercise, the glucose measurements became normal. On ultrasound scan of the kidneys she had renal structural abnormalities suggesting polycystic kidney disease. Cardiac assessment by echocardiogram was normal in both patients. The boy is currently on peritoneal dialysis for end stage renal disease; meanwhile his sister is under supervision with screening for renal deterioration, her renal function is, so far, in normal limits.

Analyzing the clinical traits of these patients we found that retinopathy, nephropathy and central obesity were present in both the Alstrom patient and his relatives with Biedl-Bardet, suggesting a main anomaly in ciliary’s function controlling photoreception, renal and metabolic processes. The patients were included in a study of linkage mapping (Jackson Laboratory, Bar Harbor, Maine). The DNA samples from the Biedl Bardet patients are currently under investigation. Interestingly, so far, the Alstrom patient doesn’t have any of the known ALMS1 mutations and he did not have any of the known BBS mutations either. He had one heterozygous SNP in BBS5 (BBS5_N184S het) that is not disease-causing. Despite the fact that this case has been analyzed for years without the identification of a known mutation, efforts are still made using the Asper Biotech Gene Chip, in order to identify a possible new unknown mutation that may explain the phenotypic variability observed in the case of his Biedl-Bardet relatives. Taking into account the clinical presentation, we still believe that the diagnosis of Alstrom is the right one for the patient despite the present absence of any identified mutations in his genome and the unexpected presence of the Bardet-Biedl cases in his family. Twelve genes are known to be associated with Bardet-Biedl syndrome. Approximately 20% to 30% of persons with BBS do not have identifiable mutations in any of the twelve known *BBS* genes; therefore, it is possible that more *BBS* genes are yet to be identified. *BBS1, BBS2, ARL6/BBS3, BBS4, MKKS/BBS6, BBS7, TTC8/BBS8, B1/BBS9, BBS10, BBS11/TRIM32 and BBS12* are of moderate size and together comprise approximately 137 exons, therefore, mutation screening for any given individual is a large undertaking. Mutations range from missense and nonsense to insertions, deletions, and splice site disruptors for most of these genes. Sequence analysis of entire exons 10 and 16 and partial exon 8 detects mutations in 25%-40% of individuals with Alstrom syndrome [Collin et al. 2002; Hearn et al. 2002; Titomanilio et al 2004; JD Marshall, GB Collin, personal communication]. In a small study of a UK population, Minton et al (2006) sequenced the entire coding region of ALMS1 and failed to identify a second mutated allele in two of 12 individuals; in two other persons (2/12) no disease-causing mutations were found [Minton et al. 2006].

**Fig. 1:**
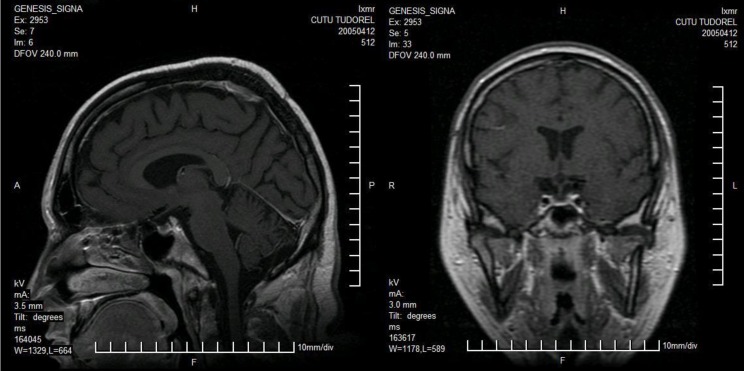
Magnetic resonance imaging – morphologic appearance suggesting “empty sella”

## Discussion

Primary cilia are ubiquitous cellular appendages that provide important yet not well understood sensory and signaling functions. Until recently, cilia were thought to be simple external cellular organelles, but now they are thought to play important roles in cell signaling, in sensing chemical and physical activity, in intracellular communication, and as photoreceptors. Cilia are microtubule-based appendages that extend from the basal bodies of cells. There are three structurally and functionally distinct types of cilia in mammals: nodal cilia, motile cilia, and primary cilia. Nodal cilia are found on cells of the embryonic node and play essential roles in establishing the left-right body axis [**3**]. Motile cilia and flagella are responsible for generating flow or movement. Primary cilia are generally immotile solitary organelles that are present on almost all human cell types [**4**, **6**]. The growth and maintenance of cilia is dependent on the bidirectional transport of proteins along their micro tubular axoneme by a process called intraflagellar transport (IFT) [**5**]. It is generally accepted that primary cilia serve important specialized signaling functions [**7**–**10**]. Photoreceptors, which are modified primary cilia, sense and respond to light. Specialized olfactory cilia detect odors and initiate signaling cascades in olfactory neurons. Primary cilia on epithelial cells in the kidney act as mechanosensors which detect and respond to fluid flow [**11**, **12**].The significance of primary cilia is exemplified by the fact that defects in cilia formation or function cause renal cystic disease, retinal degeneration, liver fibrosis, anosmia, ataxia, cardiac defects, and situs inversus [**13**, **14**]. Primary cilia also have important roles in the patterning of tissues during development [**10**, **14**], and primary cilia dysfunction is thought to underlie the etiology of numerous human genetic disorders [**15**].Yet, the specific role of primary cilia on the vast majority of cells is unknown. An increasing number of genetic diseases is associated with defects in ciliogenesis or ciliary function [**16**], including cystic kidney disease, retinal degeneration, hydrocephalus, laterality defects, chronic respiratory problems, Bardet-Biedl, Alstrom, Orofaciodigital and Meckel syndromes, cerebello-oculo-renal syndrome (Joubert syndrome type B) and Leber congenital amaurosis [**17**-**19**].

Many studies suggested an important role of G proteins in the mechanism of both BBS and Alstrom syndrome suggesting a similar molecular pathogenesis. The G protein-coupled receptors (GPCRs) somatostatin receptor 3 (Sstr3) [**20**] and serotonin receptor 6 [**21**, **22**], specifically located to neuronal cilia, suggesting a role for cilia in signaling on neurons. The functional importance of these cilia is suggested by the fact that several human cilia disorders, including Bardet-Biedl syndrome, Joubert syndrome and Meckel syndrome, have prominent functional and structural CNS phenotypes [**23**]. Pediatricians should be aware of this new category of disorders called ciliopathies. Additional multi-organ syndromes caused by cilia protein defects are likely to be identified.

**Figure 2:**
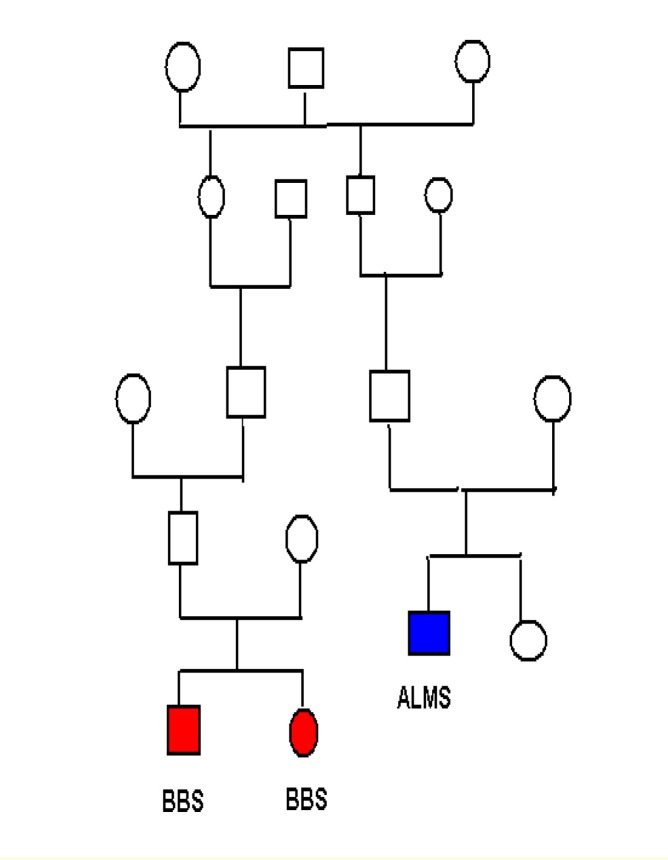
Family pedigree

## Conclusions

Although the research work in the field of this new and challenging category of disease is rapidly advancing and is bringing new arguments regarding the role of defective cilia in the genesis of syndromes like Alstrom and Bardet-Biedl, the clinical presentation and prognosis differences seen in our patients are far from being fully explained. 

How can affected siblings, even within the same family pedigree, who must share the same mutation, show such a variation in phenotypes? Age or sex alone does not seem to explain the variation. However, inheritance of different alleles of other genes may either alleviate or aggravate the expression of the disease if the product of these, currently unknown, genes interacts with the disease genes of BBS and Alstrom. The mechanism of the clinical and genetic diversity in Bardet-Biedl and Alstrom patients is not yet known. The occurrence of similar clinical cases within a family further demonstrates the existence of a common pathologic cilia mechanism, a genetic basis of phenotypic variability in seemingly monogenic disease and a functional link between rare disorders and common traits with overlapping clinical manifestations. Genetic studies in such patients may provide new data regarding the consequences of defective cilia and a possible identification of new gene mutations. Further careful clinical and genetic studies of such families with several affected individuals can contribute to a better understanding of these complex disorders.

## Acknowledgements

We thank J.D. Marshall, Genetics Coordinator Alstrom Syndrome Studies, The Jackson Laboratory, Bar Harbor, USA.

**Competing interests**

The author(s) declare that they have no competing interests.

## References

[1] Nachury MV (2007). A core complex of BBS proteins cooperates with the GTPase
Rab8 to promote cilia membrane biogenesis. Cell.

[2] (1996). Empty sella, impaired testosterone secretion, and defective hypothalamic-pituitary growth and gonad axes in children with Bardet-Biedl syndrome. Metabolism.

[3] Shiratori H, Hamada H (2006). The left-right axis in the mouse: from origin to morphology. Development.

[4] Inés Ibañez-Tallon, Nathaniel Heintz , Heymut Omran (2003). To beat or not to beat: roles of cilia in development and disease. Human Molecular Genetics.

[5] Eley L, Yates LM, Goodship JA (2005). Cilia and disease. Curr Opin Genet Dev.

[6] Wheatley DN, Wang AM, Strugnell GE (1996). Expression of primary cilia in mammalian
cells. Cell Biol Int.

[7] Pan J, Wang Q, Snell WJ (2005). Cilium-generated signaling and cilia-related disorders. Lab Invest.

[8] Singla V, Reiter JF (2006). The primary cilium as the cell’s antenna: Signaling at a sensory
organelle. Science.

[9] Marshall WF, Nonaka S (2006). Cilia: Tuning in to the cell’s antenna. Curr Biol.

[10] Eggenschwiler JT, Anderson KV (2007). Cilia and developmental signaling. Annu Rev Cell Dev Biol.

[11] Praetorius HA, Spring KR (2001). Bending the MDCK cell primary cilium increases intracellular calcium. J Membr Biol.

[12] Nauli SM (2003). Polycystins 1 and 2 mediate mechanosensation in the primary cilium of kidney cells. Nat Genet.

[13] Hildebrandt F, Otto E (2005). Cilia and centrosomes: A unifying pathogenic concept for cystic kidney disease?. Nat Rev Genet.

[14] Davenport JR, Yoder BK (2005). An incredible decade for the primary cilium: A look at a once-forgotten organelle. Am J Physiol.

[15] Fliegauf M, Benzing T, Omran H (2007). Whencilia go bad: Cilia defects and ciliopathies. Nat Rev Mol Cell Biol.

[16] Andersen J. S., Wilkinson C. J., Mayor T., Mortensen P., Nigg E. A., Mann M (2003). Proteomic characterization of the human centrosome by protein correlation profiling. Nature.

[17] Ansley S. J., Badano J. L., Blacque O. E., Hill J., Hoskins B. E., Leitch C. C., Kim J. C., Ross A. J., Eichers E. R., Teslovich T. M. (2003). Basal body dysfunction is a likely cause of pleiotropic Bardet-Biedl syndrome. Nature.

[18] Kyttala M., Tallila J., Salonen R., Kopra O., Kohlschmidt N., Paavola-Sakki P., Peltonen L., Kestila M (2006). MKS1,encoding a component of the flagella apparatus basal body proteome, is mutated in Meckel syndrome. Nat. Genet.

[19] Romio L., Fry A. M., Winyard P. J., Malcolm S., Woolf A. S., Feather S. A. (2004). OFD1 is a centrosomal/basal body protein expressed during mesenchymal-epithelial transition in human nephrogenesis. J. Am. Soc. Nephrol.

[20] Handel M (1999). Selective targeting of somatostatin receptor 3 to neuronal cilia. Neuroscience.

[21] Hamon M (1999). Antibodies and antisense oligonucleotide for probing the distribution and putative functions of central 5-HT6 receptors. Neuropsychopharmacology.

[22] Brailov I (2000). Localization of 5-HT (6) receptors at the plasma membrane of neuronal cilia in the rat brain. Brain Res.

[23] Badano JL, Mitsuma N, Beales PL, Katsanis N (2006). The ciliopathies: An emerging class of human genetic disorders. Annu Rev Genomics Hum Genet.

